# Poultry-handling Practices during Avian Influenza Outbreak, Thailand

**DOI:** 10.3201/eid1110.041267

**Published:** 2005-10

**Authors:** Sonja J. Olsen, Yongjua Laosiritaworn, Sarika Pattanasin, Prabda Prapasiri, Scott F. Dowell

**Affiliations:** * International Emerging Infections Program, Nonthaburi, Thailand; † Ministry of Public Health, Nonthaburi, Thailand

**Keywords:** avian influenza, poultry, Asia, survey, dispatch

## Abstract

With poultry outbreaks of avian influenza H5N1 continuing in Thailand, preventing human infection remains a priority. We surveyed residents of rural Thailand regarding avian influenza knowledge, attitudes, and practices. Results suggest that public education campaigns have been effective in reaching those at greatest risk, although some high-risk behavior continues.

Since January 2004, Thailand and >8 other Southeast Asian countries have experienced outbreaks of avian influenza H5N1 in poultry and >100 million poultry have been culled or died. From January 28, 2004, to February 2, 2005, Cambodia, Thailand, and Vietnam reported 55 human cases, including 42 deaths, to the World Health Organization (*1*). Twenty-four (44%) of these infections were in children <15 years of age. Although the number of human cases is small compared to poultry cases, human cases continue to occur, usually associated with close contact with sick or dying poultry. Reducing human and poultry contact is a key prevention strategy, and the Thai Ministry of Public Health has pursued an aggressive campaign to educate the Thai population on avian influenza and its prevention. To assess the effectiveness of the campaign, we carried out a survey of knowledge, attitudes, and practices regarding avian influenza in rural Thailand.

## The Study

We conducted a community cluster survey in Nakhon Phanom, a province where we have ongoing collaborative projects. A questionnaire was designed to assess knowledge, attitude, and practices before and after the interviewee had heard about avian influenza. To detect a change of >15% between results before and after the education campaign, we sampled 200 persons. From a list of villages and their populations, we selected 5 by using a probability proportional to size. The starting house in each village was preselected by randomly selecting 3 numbers between 1 and the total number of households. If no one answered at the household with the first number, the second then third numbers were tried until a starting house was identified. We interviewed persons who were >18 years of age. If >1 adult was home, the interviewer used a pregenerated random number table to determine which person to interview. Once the survey of the starting household was completed, the interviewer followed a written set of detailed instruction to find the next house. Native Thai speakers from the provincial health office were trained and conducted the interviews. Interviewers pilot-tested the survey to assess readability and comprehension, and the questionnaire was translated back into English to confirm accuracy. Interviews were conducted from August 25 to August 31, 2004, between 7:30 a.m. and 6:00 p.m. Data were analyzed by using SPSS 12.0 (SPSS Inc., Chicago, IL, USA).

During the outbreak, the Ministry of Public Health disseminated health messages on avian influenza to the public and healthcare professionals through several different types of media (Table A1). The ministry established a call-in hotline, and a frequent concern of callers was the safety of eating chicken. Call volume ranged from a minimum of 30 calls per day to a maximum of 200 calls per day. Official television messages were aired only on 3 days in February 2004. In addition, local media coverage was extensive, with daily television, newspaper, and radio reports during the peak outbreak months.

The median age of respondents was 50 years (range 22–87), and 144 (72%) of 200 were women; 122 (61%) had less than a primary school education. The median number of persons per household was 4 (range 1–11), and 110 (55%) had a child <10 years of age living in the household; 148 (74%) reported having poultry in their backyard (Table 1).

**Table 1:** Presence of poultry in 200 households, Nakhon Phanom, Thailand, 2004

Poultry	No. households (%)	Median no. poultry (range)*
Chickens	101 (51)	10 (1–85)
Fighting cocks	17 (9)	4 (1–40)
Ducks	9 (5)	7 (3–15)
Geese	2 (1)	2 (1–2)
Other†	43 (21)	Not asked
Any poultry‡	148 (74)	10 (1–100)

*Of persons reporting poultry at household. †Other species and poultry living in backyard owned by others. ‡Some households had >1 type of poultry.

All but 4 (98%) persons said they had heard of bird flu, and 179 (91%) of these said they first heard about it on the television. Only 2 (1%) respondents had seen the Ministry of Public Health Web site on avian influenza. Of the 80 persons who remembered the month they first heard about avian influenza, 51 (64%) heard about it between December 2003 and March 2004, which was during the first big outbreak. Of the people who had heard about avian influenza, 149 (76%) recognized that persons could get the disease from chicken or other poultry.

Overall, knowledge and attitudes about how to protect oneself from diseases from poultry changed significantly after the respondent heard about avian influenza (Table 2). The percentage of adults who thought touching sick or dead poultry with their bare hands was safe decreased from 40% to 14% (p<0.01) and from 23% to 5% for children in their household (p<0.01).

**Table 2:** Knowledge, attitudes, and practices before and after* hearing about avian influenza†

Variable	Before, n (%)	After, n (%)	p value
Knowledge and attitudes			
Thought it was safe to touch sick or dead poultry with bare hands	78 (40)	27 (14)	<0.01
Thought it was safe for children to touch sick or dead poultry with bare hands	45 (23)	9 (5)	<0.01
Thought it was safe to prepare raw poultry and other foods on the same cutting board	98 (50)	73 (37)	0.01
Thought it was safe to eat chicken that was pink in the middle or eggs with a runny yolk	41 (21)	11 (6)	<0.01
Practices			
Touched sick or dead poultry with bare hands	76 (39)	22 (11)	<0.01
Children in household touched sick or dead poultry with bare hands	12 (6)	7 (4)	0.4
Took dead chicken or poultry from yard and prepared it to eat	24 (12)	17 (9)	0.3
Prepared raw poultry and other foods using different cutting boards	64 (33)	83 (42)	0.08
Washed hands with water immediately after preparing raw chicken or poultry	151 (77)	158 (81)	0.3

*Participants were asked to recall the month they first heard about avian influenza and then answer questions recalling their knowledge, attitudes, and practices in the 6 months before versus the 6 months after they heard about it. †Among the 196 respondents who reported hearing about avian influenza.

In contrast, practices changed less dramatically. Touching sick or dead poultry with bare hands decreased significantly from 39% to 11% (p<0.01), but the decline was not significant for children in the household (6% to 4%, p = 0.4). Nor did a significant decline occur in the frequency of persons who reported taking dead poultry from their yard and preparing it for consumption (12% to 9%, p = 0.3). Certain practices that did not change significantly were already at somewhat appropriate levels. For example, 77% of persons reported that before they heard about avian influenza, they frequently washed their hands after touching raw poultry.

If persons were required to touch sick or dead poultry, 138 (70%) said they learned they could protect themselves by wearing gloves. Ten (5%) persons thought that wearing a mask was also protective; 172 (86%) persons believed the information they learned about how to protect themselves. When asked how much they changed their actions around poultry and poultry products since hearing about avian influenza, 38 (19%) persons said not at all, 38 (19%) said a little, 62 (32%) said a moderate amount, 47 (24%) said a lot, and 10 (5%) said completely.

## Conclusions

In Thailand, public health education campaigns and general media reports about avian influenza appear to have been effective in reaching rural people who are at greatest risk of acquiring the disease through contact with backyard poultry. However, despite widespread knowledge about avian influenza and the effective means of protection, many Thai persons have not changed their behavior. Activities such as taking dead poultry out of the backyard and preparing it for household consumption continue to put persons at increased risk. Given the continued presence of poultry outbreaks and ongoing poultry-to-human transmission, additional efforts are needed to protect humans from infection.

In contrast to the 1997 influenza H5N1 outbreak in Hong Kong, where live poultry markets were the primary source of exposure (*2*), in Thailand, human cases of avian influenza have largely resulted from contact with sick or dying poultry in the person's backyard (*3*). A case-control study of the first 12 patients with laboratory-confirmed cases of H5N1 found that contact with dead poultry was a significant risk factor for illness (*4*). Not only are poultry numerous in Southeast Asia, few birds, except those on large commercial farms, are contained by an enclosure or fence. Culling, which proved highly effective in curtailing the 1997 poultry outbreak in Hong Kong, may be a less effective control strategy in Thailand, where poultry movement is extensive and difficult to control.

Of the 1.2 million chickens and other poultry in Nakhon Phanom, 29% reside on a commercial farm with a quarantine system in place and closed indoor feeding (Nakhon Phanom Provincial Livestock Office, unpub. data). The 5 villages where the survey was conducted contained no large commercial poultry farms.

The province where our survey was conducted has never been officially declared an H5N1-affected area, although poultry die-offs and suspected human cases of avian influenza have been reported. The survey results may not be generalizable to provinces with officially declared "affected areas." However, the improvement in knowledge and attitudes seen in our province might be magnified in an affected province where people have been personally affected by chicken and human illness and deaths.

The H5N1 virus has evolved from the strain seen in Hong Kong in 1997 (*5*). The virus now has an expanded animal range, and although it does not seem well adapted to human-to-human transmission, concerns persist that this adaptation may occur. Although most human cases have been transmitted by poultry, as in the 1997 Hong Kong outbreak, Thailand recently documented limited person-to-person transmission in a family cluster (*6**–**9*).

Reducing risk by encouraging behavior change is particularly challenging and can take years. However, change is possible. For example, significant changes in sexual behavior have contributed to a decline in HIV rates in Thailand. Between the 1991 implementation of the 100% condom campaign and 1995, HIV prevalence decreased significantly in Thai military conscripts (*10*). To prevent avian influenza, changing the behavior with the highest risk, touching sick or dead poultry with bare hands, should be attempted through public education and reinforced through behavioral counseling. This message must reach children because they account for more than half of the cases of avian influenza in Thailand. If complete avoidance of sick or dead poultry is impossible, messages should include information on proper hand protection, such wearing disposable gloves or using a plastic bag, and disposal methods.

This study suggests that public campaigns can be effective at educating rural populations. Renewed efforts are needed to find practical solutions that will induce behavioral change.

## Appendix

**Table A1:** Methods of communicating public health messages used by the Thai Ministry of Public Health during the avian influenza outbreak, 2004

Type of communication	Content	Audience	Dates distributed
Telephone hotline	Prevention	General public	Jan 23–May 11
Newspaper	How to prevent avian influenza	General public	Jan 27, 28, 31
Radio	How to prevent avian influenza	General public and health professional	Jan 30–Feb 18
Website*	Surveillance, prevention	General public and health professional	Jan 30–ongoing
Television	How to prevent avian influenza	General public	Feb 10–12
Video compact disk and cassette tape	How to do surveillance for avian influenza	District health officers	Feb 10
Brochure	Transmission, symptoms, care of patients with suspected cases, prevention for cullers and food providers, safety measures taken when touching dead animals	General public	Aug

*For English version, see http://thaigcd.ddc.moph.go.th/Bird_Flu_main_en.html
